# Analysis of backward differentiation formula for nonlinear differential-algebraic equations with 2 delays

**DOI:** 10.1186/s40064-016-2422-z

**Published:** 2016-07-07

**Authors:** Leping Sun

**Affiliations:** College of Mathematics and Sciences, Shanghai Normal University, Shanghai, 200234 People’s Republic of China

**Keywords:** Stability, Backward differential formula, Delay differential-algebraic equations, Perturbations, 34A34

## Abstract

This paper is concerned with the backward differential formula or BDF methods for a class of nonlinear 2-delay differential algebraic equations. We obtain two sufficient conditions under which the methods are stable and asymptotically stable. At last, examples show that our methods are true.

## Background

For a system of differential-algebraic equations (DDAEs) (Brenan et al. 1996),

$$F(t, y(t), y'(t))=0,$$

*F* and *y* are vector valued and $\partial F/\partial y'$ may be singular. In some cases, time delays appear in variables of unknown functions so that the differential-algebraic equations (DAEs) are converted to delay differential-algebraic equations (DDAEs) (Ascher and Petzold 1995),

$$F(t, y(t), y(t-\tau ), y'(t), y'(t-\tau ))=0,$$

where *F* and *y* are vector valued, $\tau >0$ is a constant, $\partial F/\partial y'$ may be singular. If $y'(t-\tau )$ does not vanish, it is actually called neutral delay differential-algebraic equations (NDDAEs), otherwise it is called delay differential-algebraic equations (DDADs). In 1995, authors in Ascher and Petzold (1995) discussed the convergence of BDF methods and Runge–Kutta methods solving initial-value differential-algebraic equations of retarded and neutral types, corresponding to the structure of Hessenberg forms; in 1997, authors Zhu and Petzold (1997) considered the asymptotic stability of linear constant coefficient differential-algebraic equations and obtained numerical results on *θ*-methods, Runge–Kutta methods and linear multistep methods to these systems. In 1998, Zhu and Petzold (1998) got further results on stability of Hessenberg DDAEs of retarded or neutral type. In 2005, stability of Rosenbrock methods for neutral delay differential-algebraic equatuons was discussed in Zhao and Xu (2005). Earlier, authors of Torelli (1989), Mechee et al. (2013) were interested in the numerical treatments on delay differential equations which are delay differential-algebraic equations with $\partial F/\partial y'$ nonsingular. Authors in Fan et al. (2013), Liu et al. (2014) gave criteria for stability of neutral delay differential-algebraic equations geometrically and obtained stable regions over which numerical methods could be used effectively. Among these results, there are few achievements on nonlinear systems. In fact, the solution of nonlinear system depends on a nonlinear manifold of a product space and on consistent initial valued-vectors over a space of continuous functions so that research on nonlinear DDAEs is more complicated and still remains investigated.

Authors in Kuang and Cong (2005), Ascher and Petzold (1998) denote that numerical approaches for the solution of differential-algebraic equations (DAEs) can be divided roughly into two classes. One is direct discretizations of the given system, the other is involving a reformulation, combined with a discretization. Practically all the winning methods have stiff decay. For initial value DAEs which are cumbersome and especially for DAEs whose underlying ODEs are stiff, the backward differentiation formulae (BDF) and Radau collocation methods are the overall methods of choice.

In this paper, we investigate a class of nonlinear DDAE system, and show the conditions under which two-step BDF methods are stable and asymptotically stable.

## Asymptotic behavior of 2-delay differential-algebraic equations

Now we consider the following nonlinear system of delay differential-algebraic equations,

1 $$u'(t)= f(t, u(t), u(t-\tau ), v(t), v(t-\tau )),\quad t>0, (\tau >0)$$

2 $$0= \varphi (u(t), u(t-\tau ), v(t)),\quad t>0,$$

According to Ascher and Petzold (1995) the assumption that $\varphi _{v}$ is nonsingular allows one to solve the constraint equations (2) for *v*(*t*) using the implicit theorem, yielding

3 $$v(t)= g(u(t), u(t-\tau )),\quad t>0,$$

by substituting (3) into (1) we obtain the DODE

4 $$u'(t)= f(t,u(t), u(t-\tau ), g(u(t), u(t-\tau ), u(t-2\tau ))),$$

Thus, the DDAEs (1) and (2) are stable if the DODE (4) is stable. Note that if all the delay terms are present in this retarded DODE, then the initial conditions need to be defined for *t* on $[-2\tau , 0]$. So in fact, we will investigate (1) and (2) by following nonlinear system of delay differential-algebraic equations,

5 $$u'(t)= f(t, u(t), u(t-\tau ),v(t), v(t-\tau )),\quad t>0, (\tau >0)$$

6 $$0= \varphi (u(t), u(t-\tau ), v(t)),\quad t>0,$$

7 $$u(t)= \varphi _{1}(t), v(t)=\psi _{1}(t),\quad -\tau \le t \le 0,$$

8 $$u(t)= \varphi _{2}(t), v(t)=\psi _{2}(t), \quad -2\tau \le t\le -\tau ,$$

and its perturbed equations

9 $$\tilde{u}'(t)= f(t, \tilde{u}(t), \tilde{u}(t-\tau ), \tilde{v}(t), \tilde{v}(t-\tau )), \quad t> 0,(\tau >0)$$

10 $$0= \varphi (\tilde{u}(t), \tilde{u}(t-\tau ), \tilde{v}(t)), \quad t>0,$$

11 $$\tilde{u}(t)= \tilde{\varphi }_{1}(t), \tilde{v}(t)=\tilde{\psi }_{1}(t),\quad -\tau \le t \le 0,$$

12 $$\tilde{u}(t)= \tilde{\varphi }_{2}(t), \tilde{v}(t)=\tilde{\psi }_{2}(t),\quad -2\tau \le t\le -\tau ,$$

From results of Torelli (1989), we hope the estimations on $u(t)-\tilde{u}(t)$ and $v(t)-\tilde{v}(t)$ satisfy

$$\begin{aligned}&\Vert u(t)-\tilde{u}(t)\Vert \le \max _{-\tau \le t \le 0}\Vert \Phi (t)-\tilde{\Phi }(t)\Vert , \quad \forall t \ge 0,\\&\Vert v(t)-\tilde{v}(t)\Vert \le \max _{-\tau \le t \le 0}\Vert \Psi (t)-\tilde{\Psi }(t)\Vert , \quad \forall t \ge 0, \end{aligned}$$

In practice, the following definition is to be considered.

### **Definition 1**

Liu et al. (2014) System (1)–(2) is said to be stable, if the follow inequalities are satisfied,

13 $$\Vert u(t)-\tilde{u}(t)\Vert\le \max _{-2\tau \le t \le 0}\Vert \Phi (t)-\tilde{\Phi }(t)\Vert ,$$

14 $$\Vert v(t)-\tilde{v}(t)\Vert\le M\max _{-2\tau \le t \le 0}\Vert \Phi (t)-\tilde{\Phi }(t)\Vert ,$$

where *M* > 0 is a constant,

15 $$\begin{aligned} \Phi ={\left\{ \begin{array}{ll}\varphi _{1}(t), & \quad -\tau \le t \le 0, \\ \varphi _{2}(t), & \quad -2\tau \le t \le -\tau , \end{array}\right. }\quad \tilde{\Phi }={\left\{ \begin{array}{ll}\tilde{\varphi }_{1}(t), & \quad -\tau \le t \le 0, \\ \tilde{\varphi }_{2}(t), & \quad -2\tau \le t \le -\tau . \end{array}\right. } \end{aligned}$$

To study the stability of DDAE (1)–(2), we can investigate equations (5)–(8) and the perturbations (9)–(12). Ascher and Petzold (1995) showed that under some conditions the analytical solutions of the system is stable and asymptotically stable. In the next section, we will discuss the stability behavior of 2-step BDF methods for a class of the system based on the assumption that the analytical solution exists uniquely and stable.

## The stability and asymptotic stability of 2-step BDF methods

Firstly, the 2-step BDF methods are introduced as follows.

### Backward differentiation formula

For the differential equation

$$y'(t)=f(t,y),$$

the Backward Differentiation Formula or BDF methods are derived by differentiating the polynomial which interpolates past values of y, each step is h, and setting the derivative at $t_{n}$ to $f(t_{n},y_{n})$. This yields the *k*-step BDF, which has order *p* = *k*,

$$\sum\limits_{{i = 1}}^{k} {\frac{1}{i}} \nabla ^{i} y_{n} = hf(t_{n} ,y_{n} ),$$

this can be written in scaled form where $\alpha _{0}=1$,

$$\sum _{i=1}^k\alpha _{i}y_{n-i}=h\beta _{0}f(t_{n},y_{n}),$$

here we apply 2-step BDF, the formula can be written as

$$y_{n}-\frac{4}{3}y_{n-1}+\frac{1}{3}y_{n-2}=\frac{2}{3}hf(t_{n},y_{n}),$$

For the initial value problem of the ordinary differential equations

16 $$x'(t)= f(t,x(t)), \quad t>0,$$

17 $$x(0)= x_{0},$$

The 2-setp BDF methods can be written as:

18 $$x_{n+2}= \frac{4}{3}x_{n+1}-\frac{1}{3}x_{n}+\frac{2}{3}h f(t_{n+2},x_{n+2}),\quad n=0,1,2,\ldots ,$$

19 $$x_{0}= x(0),$$

where $x_{n}\sim x(t_{n})$, $h>0$ is the step size. To solve (5)–(8) and (9)–(12) by (18)–(19), we get

20 $$u_{n+2}= \frac{4}{3}u_{n+1}-\frac{1}{3}u_{n}+\frac{2}{3}h f(t_{n+2},u_{n+2},u_{n+2-m},v_{n+2},v_{n+2-m}),\quad n=0,1,2,\ldots ,$$

21 $$0= \varphi (u_{n+1},u_{n+1-m},v_{n+1}),$$

22 $$u_{n}= \varphi _{1}(t_{n}),v_{n}=\psi _{1}(t_{n}), \quad -m\le n\le 0,$$

23 $$u_{n}= \varphi _{2}(t_{n}),v_{n}=\psi _{2}(t_{n}), -2m\le n\le -m,\quad (mh=\tau , m\ge 1).$$

The perturbations of (20)–(21) are

24 $$\begin{aligned} \tilde{u}_{n+2}&= \frac{4}{3}\tilde{u}_{n+1}-\frac{1}{3}\tilde{u}_{n}+\frac{2}{3}h f(t_{n+2},\tilde{u}_{n+2},\tilde{u}_{n+2-m},\tilde{v}_{n+2},\tilde{v}_{n+2-m}),\quad n=0,1,2,\ldots , \end{aligned}$$

25 $$0= \varphi (\tilde{u}_{n+1},\tilde{u}_{n+1-m},\tilde{v}_{n+1}),$$

26 $$\tilde{u}_{n}= \tilde{\varphi }_{1}(t_{n}),\tilde{v}_{n}=\tilde{\psi }_{1}(t_{n}), \quad -m\le n\le 0,$$

27 $$\tilde{u}_{n}= \tilde{\varphi }_{2}(t_{n}),\tilde{v}_{n}=\tilde{\psi }_{2}(t_{n}), -2m\le n\le -m,\quad (mh=\tau , m\ge 1).$$

If the step size is $h>0$ and $t_{n}=nh$ and the numerical approximations are $u_{n}\approx u(t_{n})$, it should be note that $t_{i}-\tau$ may not be a grid point $t_{j}$ for any *j*. Then a function interpolation is needed so that

$$\begin{aligned} u_{n+i-m}=\delta _{u} u_{n+i+1-m}+(1-\delta _{u})u_{n+i-m},\quad v_{n+j-m}=\delta _{v} v_{n+j+1-m}+(1-\delta _{v})v_{n+j-m}, \end{aligned}$$

here $0<\delta _{u}, \delta _{v}<1$, the convergence order of interpolation is 2 and the local truncation error of the method is 3, then the convergence order of the iteration by BDF method is two (Kuang and Cong 2005). For simplicity, we just consider $u_{n+i-m}, v_{n+j-m}$ are on grid points or obtained by interpolations.

### The stability of 2-step BDF methods

Let $u_{\tau }=u(t-\tau ), v_{\tau }=v(t-\tau )$. We require that *f*, $\varphi$ in (5), (6), (9), and (10) satisfy the following Lipschitz conditions (1)–(4):

1. $$\langle f(t,u,u_{\tau },v,v_{\tau })-f(t,\tilde{u},u_{\tau },v,v_{\tau }), u-\tilde{u}\rangle \le \sigma (t)\Vert u-\tilde{u}\Vert ^{2},$$
2. $$\begin{aligned}&\Vert f(t,u,u_{\tau },v,v_{\tau })-f(t,u,\tilde{u}_{\tau },v,v_{\tau })\Vert \le \gamma _{1}(t)\Vert u_{\tau }-\tilde{u}_{\tau }\Vert ,\\&\Vert f(t,u,u_{\tau },v,v_{\tau })-f(t,u,u_{\tau },\tilde{v},v_{\tau })\Vert \le \gamma _{2}(t)\Vert v-\tilde{v}\Vert ,\\&\Vert f(t,u,u_{\tau },v,v_{\tau })-f(t,u,u_{\tau },v,\tilde{v}_{\tau })\Vert \le \gamma _{3}(t)\Vert v_{\tau }-\tilde{v}_{\tau }\Vert , \end{aligned}$$
3. $\varphi _{v}$ is nonsingular, so that for *g*(*u*, *v*) in (3), there exist $L>0$, $K>0$, such that
  $$\begin{aligned}&\Vert g(u, v)-g(\tilde{u}, v)\Vert \le L\Vert u-\tilde{u}\Vert ,\quad \Vert g(u, v)-g(u, \tilde{v})\Vert \le K\Vert v-\tilde{v}\Vert ,\\&\sigma (t)<0,\quad \frac{1}{2}\sigma _{1}(t)+\gamma _{1}(t)+(L+K)\gamma _{2}(t)+(L+K)\gamma _{3}(t)\le -\sigma (t),\quad t>0, \end{aligned}$$
  where $\sigma _{1}(t)$ is an increasing function described in the following Theorem 1.
  Note: $\sigma _(t)<0$ means the right side of function in condition (1) is negative, examples in the last section show the situation exists.
4. The Frechet derivatives of *g*(*u*, *v*) with regard to *u*, *v*, $\frac{\partial g}{\partial u}$, $\frac{\partial g}{\partial v}$ exist in the product space $\mathbb {R}^{d}\times \mathbb {R}^{d}$, $\frac{\partial g}{\partial v}$ is continuous, $(\frac{\partial g}{\partial u})^{-1}$ exists, and
  $$\begin{aligned} \sup _{u,v\in \mathbb {R}^{d}}\left\| \left( \frac{\partial g}{\partial v}\right) ^{-1}\left( \frac{\partial g}{\partial u}\right) \right\| =L<\infty , \end{aligned}$$
  where $$u=(u_{1},u_{2},\ldots ,u_{d})^{T}$$, $$v=(v_{1},v_{2},\ldots ,v_{d})^{T}$$, $$\langle u,v\rangle =\sum ^{d}_{i=1}u_{i}v_{i}$$, $$\Vert u\Vert ^{2}=\langle u,u\rangle$$,
  $$\left\| \frac{\partial g}{\partial u}\right\| =\sup _{\omega \in \mathbb {R}^{d}, \Vert \omega \Vert =1}\left\| \left( \frac{\partial g}{\partial u}\right) \omega \right\| .$$

Here $|\sigma (t)|, \sigma _{1}(t), \gamma _{i}(t),i=1,2,3, t>0$ are increasing functions defined on time. The Frechet derivatives are described as follows, If

$$\begin{aligned} x&=(x_{1},x_{2}, \ldots , x_{n})^{T}\in \mathbb {R}^{n},\\ F(x)&=(f_{1}(x),f_{2}(x),\ldots ,f_{m}(x))^{T}\in \mathbb {R}^{m},\\ f_{j}(x)&=f_{j}(x_{1},x_{2},\ldots ,x_{n})\in \mathbb {R},\quad j=1,\ldots ,m, \end{aligned}$$

$f_{j}(x)\;(j=1,2,\ldots ,m)$ has first-order continuous partial derivative at $x=x_{0}$, then the Frechet derivative $F'(x)$ can be expressed by the following matrix:

$$F'(x_{0})=\left( \begin{array}{cccc} \frac{\partial f_{1}}{\partial x_{1}} &\quad \frac{\partial f_{1}}{\partial x_{2}} &\quad \cdots & \frac{\partial f_{1}}{\partial x_{n}} \\ \frac{\partial f_{2}}{\partial x_{1}} &\quad \frac{\partial f_{2}}{\partial x_{2}} &\quad \cdots &\quad \frac{\partial f_{2}}{\partial x_{n}} \\ \vdots &\quad \vdots &\quad \vdots &\quad \vdots \\ \frac{\partial f_{m}}{\partial x_{1}} &\quad \frac{\partial f_{m}}{\partial x_{2}} &\quad \cdots &\quad \frac{\partial f_{m}}{\partial x_{n}} \\ \end{array} \right) _{x=x_{0}}$$

#### **Definition 2**

A numerical method for solving DDAEs is called stable, if for every consistent initial value functions $\Phi$, $\tilde{\Phi }$, and each step $h>0$, the solution sequences $\{u_{n}, v_{n}\}$, $\{\tilde{u}_{n}, \tilde{v}_{n}\}$ for (5)–(8) and (9)–(12) in which $f, \varphi$ satisfy conditions (1)–(4), satisfy

$$\begin{aligned}&\Vert u_{n}-\tilde{u}_{n}\Vert \le \max _{-2\tau \le t\le 0}\Vert \Phi (t)-\tilde{\Phi }(t)\Vert ,\quad n=0,1,2, \ldots , \\&\Vert v_{n}-\tilde{v}_{n}\Vert \le M\max _{-2\tau \le t\le 0}\Vert \Phi (t)-\tilde{\Phi }(t)\Vert ,\quad n=0,1,2, \ldots , \end{aligned}$$

for some $M>0$. Now the sufficient condition with which the DDAEs are stable is as follows.

#### **Theorem 1**

*The 2-step BDF methods are stable for DDAEs if*$f, \varphi$*satisfy conditions (1)–(4) and*

$$\Vert f(t,u,u_{\tau },v,v_{\tau })-f(t,\tilde{u},\tilde{u}_{\tau },\tilde{v}, \tilde{v}_{\tau })\Vert \le \sigma _{1}(t)\Vert u-\tilde{u}\Vert ,$$

*Note: it seems more natural if*$\Vert f(t,u,u_{\tau },v,v_{\tau })-f(t,\tilde{u},u_{\tau },v,v_{\tau }) \Vert \le \sigma _{1}(t)\Vert u-\tilde{u}\Vert$*is true, but we find proofs are analogous with this condition but only cumbersome and results are true without this assumption throughout the discussion in this paper.*

#### *Proof*

Let $\bar{V}_{n}=u_{n}-\tilde{u}_{n}$. Substituted into (20) and (24),

28 $$\begin{aligned} \bar{V}_{n+2}&=\frac{4}{3}\bar{V}_{n+1}-\frac{1}{3}\bar{V}_{n}+\frac{2}{3}h\{f(t_{n+2},u_{n+2},u_{n+2-m},v_{n+2},v_{n+2-m})\\&\quad -f(t_{n+1},\tilde{u}_{n+2},\tilde{u}_{n+2-m},\tilde{v}_{n+2},\tilde{v}_{n+2-m})\}\\&=\bar{V}_{n+1}+\frac{1}{3}(\bar{V}_{n+1}-\bar{V}_{n})+\frac{2}{3}h\{f(t_{n+2},u_{n+2},u_{n+2-m},v_{n+2},v_{n+2-m})\\&\quad -f(t_{n+2},\tilde{u}_{n+2},u_{n+2-m},v_{n+2},v_{n+2-m})+f(t_{n+2},\tilde{u}_{n+2},u_{n+2-m},v_{n+2},v_{n+2-m})\\&\quad -f(t_{n+2},\tilde{u}_{n+2},\tilde{u}_{n+2-m},v_{n+2},v_{n+2-m})+f(t_{n+2},\tilde{u}_{n+2},\tilde{u}_{n+2-m},v_{n+2},v_{n+2-m})\\&\quad -f(t_{n+2},\tilde{u}_{n+2},\tilde{u}_{n+2-m},\tilde{v}_{n+2},v_{n+2-m})+f(t_{n+2},\tilde{u}_{n+2},\tilde{u}_{n+2-m},\tilde{v}_{n+2},v_{n+2-m})\\&\quad -f(t_{n+2},\tilde{u}_{n+2},\tilde{u}_{n+2-m},\tilde{v}_{n+2},\tilde{v}_{n+2-m}\}. \end{aligned}$$

An inner product of (28) with $\bar{V}_{n+1}=u_{n+1}-\tilde{u}_{n+1},$

$$\begin{aligned} \left\langle \bar{V}_{n+2}, \bar{V}_{n+2}\right\rangle&=\left\langle \bar{V}_{n+2}, \bar{V}_{n+1}\right\rangle +\frac{1}{3}\left\langle \bar{V}_{n+1}-\bar{V}_{n}, \bar{V}_{n+2}\right\rangle \\&\quad +\frac{2}{3}h\langle f(t_{n+2},u_{n+2},u_{n+2-m},v_{n+2},v_{n+2-m})\\&\quad -f(t_{n+1},\tilde{u}_{n+2},\tilde{u}_{n+2-m},\tilde{v}_{n+2},\tilde{v}_{n+2-m}), \bar{V}_{n+2} \rangle , \end{aligned}$$

apply Schwartz theorem and condition (1)–(2), we obtain

29 $$\begin{aligned} \Vert \bar{V}_{n+2}\Vert ^{2}&\le \Vert \bar{V}_{n+1}\Vert \Vert \bar{V}_{n+2}\Vert +\frac{1}{3}\Vert \bar{V}_{n+1}-\bar{V}_{n}\Vert \Vert \bar{V}_{n+2}\Vert +\frac{2}{3}h(\sigma (t_{n+2})\Vert \bar{V}_{n+2}\Vert ^{2}\\&\quad +\gamma _{1}(t_{n+2})\Vert \bar{V}_{n+2-m}\Vert \cdot \Vert \bar{V}_{n+2}\Vert +\gamma _{2}(t_{n+2})\Vert v_{n+2}-\tilde{v}_{n+2}\Vert \cdot \Vert \bar{V}_{n+2}\Vert \\&\quad +\gamma _{3}(t_{n+2})\Vert v_{n+2-m}-\tilde{v}_{n+2-m}\Vert \cdot \Vert \bar{V}_{n+2}\Vert ). \end{aligned}$$

Assume that $\Vert \bar{V}_{n+2}\Vert \ne 0$ (otherwise no perturbations), note (3) and condition (3), (4), we conclude

30 $$\begin{aligned}&\Vert v_{n+2}-\tilde{v}_{n+2}\Vert \le L\Vert u_{n+2}-\tilde{u}_{n+2}\Vert +K\Vert u_{n+2-m}-\tilde{u}_{n+2-m}\Vert ,\\&\Vert v_{n+2-m}-\tilde{v}_{n+2-m}\Vert \le L\Vert u_{n+2-m}-\tilde{u}_{n+2-m}\Vert +K\Vert u_{n+2-2m}-\tilde{u}_{n+2-2m}\Vert ,\\&n=0,1,2,\ldots , \end{aligned}$$

(29) divided by $\Vert \bar{V}_{n+2}\Vert$, and note the consistency of the initial value function, we get

31 $$\begin{aligned} \Vert \bar{V}_{n+2}\Vert \le \frac{\Vert \bar{V}_{n+1}\Vert +\frac{1}{3}\Vert \bar{V}_{n+1}-\bar{V}_{n}\Vert +\frac{2}{3}h\omega (t_{n+2})\Vert \bar{V}_{n+2-m}\Vert +\frac{2}{3}hK\gamma _{3}(t_{n+2})\Vert \bar{V}_{n+2-2m}\Vert }{1-\frac{2}{3}h(\sigma (t_{n+2})+L\gamma _{2}(t_{n+2}))}, \end{aligned}$$

where $\omega (t_{n+2})=\gamma _{1}(t_{n+2})+K\gamma _{2}(t_{n+2})+L\gamma _{3}(t_{n+2}), n=0, 1, 2, \ldots , \quad \bar{V}_{0}, \bar{V}_{1}$ are two initial values for 2-step BDF methods where $\Vert V_{0}\Vert \le \max \limits _{-2\tau \le t\le 0}\Vert \Phi (t)-\tilde{\Phi }(t)\Vert$, $\bar{V}_{1}$ is evaluated by using Implicit Euler method

32 $$\Vert \bar{V}_{1}-\bar{V}_{0}\Vert =h\Vert f(t_{1},u_{1},u_{1-m},v_{1},v_{1-m}) -f(\tilde{t}_{1},\tilde{u}_{1},\tilde{u}_{1-m},\tilde{v}_{1},\tilde{v}_{1-m})\Vert$$

and conditions (1)–(3), by a simple induction, we get

33 $$\Vert \bar{V}_{1}\Vert \le \max \limits _{-2\tau \le t\le 0}\Vert \Phi (t)-\tilde{\Phi }(t)\Vert ,$$

and with the condition of this theorem, yields

34 $$\Vert \bar{V}_{1}-\bar{V}_{0}\Vert \le h\sigma _{1}(t_{1})\Vert u_{1}-\tilde{u}_{1}\Vert =h\sigma _{1}(t_{1})\Vert \bar{V}_{1}\Vert ,$$

hence, as $$n=0$$,

35 $$\begin{aligned} \Vert \bar{V}_{2}\Vert \le \frac{\Vert \bar{V}_{1}\Vert +\frac{1}{3}h\sigma _{1}(t_{1})\Vert \bar{V}_{1}\Vert +\frac{2}{3}h\omega (t_{2})\Vert \bar{V}_{2-m}\Vert +\frac{2}{3}hK\gamma _{3}(t_{2}) \Vert \bar{V}_{2-2m}\Vert }{1-\frac{1}{3}h(\sigma (t_{2})+L\gamma _{2}(t_{2}))}, \end{aligned}$$

with condition (3) and (33) and the incretion of $|\sigma (t)|, \sigma _{1}(t), \gamma _{i}(t),i=1,2,3$, we get

36 $$\Vert V_{2}\Vert \le \max \limits _{-2\tau \le t\le 0}\Vert \Phi (t)-\tilde{\Phi }(t)\Vert ,$$

as $n=1$, we evaluate $\Vert \bar{V}_{3}\Vert$ in (31) in terms of $\Vert \bar{V}_{2}\Vert , \Vert \bar{V}_{2}-\bar{V}_{1}\Vert$ in the following.

$$\begin{aligned} \bar{V}_{2}=\bar{V}_{1}+\frac{1}{3}(\bar{V}_{1}-\bar{V}_{0})+\frac{2}{3} h(f(t_{2},u_{2},{u}_{2-m},v_{2},{v}_{2-m}) -f(t_{2},\tilde{u}_{2},\tilde{u}_{2-m},\tilde{v}_{2},\tilde{v}_{2-m})), \end{aligned}$$

then, from condition (1) and (34)

37 $$\begin{aligned} \Vert \bar{V}_{2}-\bar{V}_{1}\Vert&\le \frac{1}{3}\Vert \bar{V}_{1}-\bar{V}_{0}\Vert +\frac{2}{3}h\Vert (f(t_{2},u_{2},{u}_{2-m},v_{2},{v}_{2-m})\\&\quad -f(t_{2},\tilde{u}_{2},\tilde{u}_{2-m},\tilde{v}_{2},\tilde{v}_{2-m})\Vert \\&\le \frac{1}{3}h\sigma _{1}(t_{1})\Vert \bar{V}_{1}\Vert +\frac{2}{3}h\sigma _{1}(t_{2}) \Vert \bar{V}_{2}\Vert \\&\le h\sigma _{1}(t_{2})(\frac{1}{3}\Vert \bar{V}_{1}\Vert +\frac{2}{3}\Vert \bar{V}_{2}\Vert ), \end{aligned}$$

substitute (37) into (31), take *n* = 2, also note the incretion of $|\sigma (t)|, \sigma _{1}(t), \gamma _{i}(t),i=1,2,3$, we get the estimation of $\Vert \bar{V}_{3}\Vert$ in the following,

38 $$\begin{aligned} \Vert \bar{V}_{3}\Vert \le \frac{\Vert \bar{V}_{2}\Vert +\frac{1}{3}h\sigma _{1}(t_{3})\left( \frac{1}{3}\Vert \bar{V}_{1}\Vert +\frac{2}{3}\Vert \bar{V}_{2}\Vert \right) +\frac{2}{3}h\omega (t_{3})\Vert \bar{V}_{3-m}\Vert +\frac{2}{3}hK\gamma _{3}(t_{3})\Vert \bar{V}_{3-2m}\Vert }{1-\frac{2}{3}h(\sigma (t_{3})+L\gamma _{2}(t_{3}))}, \end{aligned}$$

note condition (3), (33) and (36), $\bar{V}_{3-m}, \bar{V}_{3-2m}$ are initial valued functions with (33) or (36) satisfied too, so we get

$$\Vert V_{3}\Vert \le \max \limits _{-2\tau \le t\le 0}\Vert \Phi (t)-\tilde{\Phi }(t)\Vert ,$$

similarly, when *n* = 2, 3, 4,…, by iteration,

$$\Vert V_{n}\Vert \le \max \limits _{-2\tau \le t\le 0}\Vert \Phi (t)-\tilde{\Phi }(t)\Vert ,$$

applying mathematical induction, we conclude it is true for all $n\ge 0$. As for $\Vert v_{n}-\tilde{v}_{n}\Vert$, just see (28)

$$\Vert v_{n}-\tilde{v}_{n}\Vert \le M\max _{-2\tau \le t\le 0}\Vert \Phi (t)-\tilde{\Phi }(t)\Vert .$$

$$\square$$

### The asymptotic stability of 2-step BDF methods

Now we give the following definition.

#### **Definition 3**

The delay differential-algebraic equations (5)–(8) are asymptotically stable if and only if for every consistent initial value functions $\Phi (t)$, $\tilde{\Phi }(t)$, solutions $\{u(t), v(t)\}$, $\{\tilde{u}(t), \tilde{v}(t)\}$ satisfy

$$\begin{aligned}&\lim _{t\rightarrow \infty }\Vert u(t)-\tilde{u}(t)\Vert =0, \\&\lim _{t\rightarrow \infty }\Vert v(t)-\tilde{v}(t)\Vert =0, \end{aligned}$$

#### **Theorem 2**

*If*$f, \varphi$*satisfy conditions (1)–(4) and the following* ($3'$)

$$\begin{aligned} \sigma (t)+L\gamma _{2}(t)\le -\beta<0,\quad \sup \limits _{t\ge 0}\frac{\frac{1}{3}\sigma _{1}(t)+\frac{2}{3}(\gamma _{1}(t) +K\gamma _{2}(t)+(L+K)\gamma _{3}(t))}{-\frac{2}{3}(\sigma (t)+L\gamma _{2}(t))}=q,\quad 0 \le q<1. \end{aligned}$$

*Then the 2-step BDF methods are asymptotically stable for DDAEs Here*$|\sigma (t)|, \sigma _{1}(t), \gamma _{i}(t),i=1,2,3, t\ge 0$*are increasing functions. Note: The system is stable if*$q=1$*whileqstrictly less than 1 is required for asymptotic stability*.

#### *Proof*

Let $V_{n}=\Vert u_{n}-\tilde{u}_{n}\Vert$, from (31) (35) (38), we have

39 $$\begin{aligned}&\Vert \bar{V}_{n+2}\Vert \\&\quad \le \frac{\Vert \bar{V}_{n+1}\Vert +\frac{1}{3}h\sigma _{1}(t_{n+1})\left( \frac{1}{3}\Vert \bar{V}_{n}\Vert +\frac{2}{3}\Vert \bar{V}_{n+1}\Vert \right) +\frac{2}{3}h\omega (t_{n+2})\Vert \bar{V}_{n+2-m}\Vert +\frac{2}{3}hK\gamma _{3} (t_{n+2})\Vert \bar{V}_{n+2-2m}\Vert }{1-\frac{2}{3}h(\sigma (t_{n+2})+L\gamma _{2}(t_{n+2}))}. \end{aligned}$$

Let $0\le n\le 2m-1$ in the above inequality, we get

$$\begin{aligned} \Vert \bar{V}_{n+2}\Vert \le \max \limits _{t\ge 0}\frac{1+\frac{1}{3}h\sigma _{1}(t_{n+2})+\frac{2}{3}h(\omega (t_{n+2})+K\gamma _{3}(t_{n+2}))}{1-\frac{2}{3}h(\sigma (t_{n+2})+L\gamma _{2}(t_{n+2}))}\cdot \max \limits _{-2\tau \le t\le 0}\Vert \Phi (t)-\tilde{\Phi }(t)\Vert . \end{aligned}$$

Note condition (3′), there is $0<p<1$ such that

$$\begin{aligned} \frac{1+\frac{1}{3}h\sigma _{1}(t)+\frac{2}{3}h(\omega (t)+K\gamma _{3}(t))}{1+\frac{2}{3}h|\sigma (t)+L\gamma _{2}(t)|} \le \frac{1+\frac{2}{3}hq|\sigma (t)+L\gamma _{2}(t)|}{1+\frac{2}{3}h|\sigma (t)+L\gamma _{2}(t)|}\le \frac{1+\frac{2}{3}h\beta q}{1+\frac{2}{3}h\beta }=p<1. \end{aligned}$$

Therefore, when $$0 \le n\le 2m-1$$

$$\Vert \bar{V}_{n+2}\Vert \le p\max \limits _{-2\tau \le t\le 0}\Vert \Phi (t)-\tilde{\Phi }(t)\Vert .$$

For the case $n=2m$

$$\begin{aligned}&\Vert \bar{V}_{2m+2}\Vert \\&\quad \le \frac{\Vert \bar{V}_{2m+1}\Vert +\frac{1}{3}h\sigma _{1}(t_{2m+1})\left( \frac{1}{3}\Vert \bar{V}_{2m}\Vert +\frac{2}{3}\Vert \bar{V}_{2m+1}\Vert \right) +\frac{2}{3}h\omega (t_{2m+2})\Vert \bar{V}_{m+2}\Vert +\frac{2}{3}hK\gamma _{3}(t_{2m+2})\Vert \bar{V}_{2}\Vert }{1-\frac{2}{3}h(\sigma (t_{2m+2})+L\gamma _{2}(t_{2m+2}))}.\\ \end{aligned}$$

As indicated above,

$$\Vert \bar{V}_{2m+2}\Vert \le p^{2}\max \limits _{-2\tau \le t\le 0}\Vert \Phi (t)-\tilde{\Phi }(t)\Vert .$$

For the case $2rm\le n \le 2(r+1)m-1$, it can be shown by induction that

$$\Vert \bar{V}_{n+2}\Vert \le p^{r+1}\max \limits _{-2\tau \le t\le 0}\Vert \Phi (t)-\tilde{\Phi }(t)\Vert .$$

When $$r\rightarrow \infty ,\quad n\rightarrow \infty$$

$$\Vert \bar{V}_{n+2}\Vert \rightarrow 0,\quad (n\rightarrow \infty )$$

Thus,

$$\Vert u_{n}-\tilde{u}_{n}\Vert \rightarrow 0,\quad \Vert v_{n}-\tilde{v}_{n}\Vert \rightarrow 0,\quad (n\rightarrow \infty ).$$

$$\square$$

## Numerical examples

First, we give an example for Theorem 1.

### *Example 1*

Let *u*(*t*), $$v(t)\in \mathbb {R}$$,   $$f:\mathbb {R}\times \mathbb {R}\times \mathbb {R}\times \mathbb {R}\times \mathbb {R}\rightarrow \mathbb {R}$$,   $$g:\mathbb {R}\times \mathbb {R}\rightarrow \mathbb {R}$$.

40 $$u'(t)= \sigma (t)u(t)+P_{1}(t)f_{1}(u(t-\tau ))+P_{2}(t)f_{2}(v(t))+P_{3}(t)f_{3} (v(t-\tau ))$$

41 $$0= g(u,v)$$

where $\sigma (t)$, $\sigma _{1}(t)$, $P_{1}(t)$, $P_{2}(t)$, $P_{3}(t)$ are polynomials of *t*, $u(t-\tau )=0$. Condition (1)–(4) say if,

42 $$\mathop {\sup }\limits_{{u,v \in \mathbb{R}}} \left| {\left( {\left. {\frac{{\partial g}}{{\partial v}}} \right)^{{ - 1}} \left( {\frac{{\partial g}}{{\partial u}}} \right.} \right)} \right| \le L < \infty .$$

43 $$\gamma _{i}(t)=|P_{i}(t)|,\quad i=1,2,3$$

44 $$|f_{i}(u)-f_{i}(\tilde{u})|\le L_{i}|u-\tilde{u}|,\quad i=1,2,3$$

45 $$\sigma (t)<0, \quad \frac{1}{2}\sigma _{1}(t)+L_{1}\gamma _{1}(t)+L(L_{2}\gamma _{2}(t)+L_{3} \gamma _{3}(t))\le -\sigma (t),$$

then (40)–(41) is stable. In fact,

1. $$\begin{aligned}&\langle f(t,u,u_{\tau },v,v_{\tau })-f(t,\tilde{u},u_{\tau },v,v_{\tau }),u-\tilde{u}\rangle =\sigma (t)|u-\tilde{u}|^{2},\\&|f(t,u,u_{\tau },v,v_{\tau })-f(t,\tilde{u},\tilde{u}_{\tau },\tilde{v},\tilde{v}_{\tau })|\le \sigma _{1}(t)|u-\tilde{u}| \end{aligned}$$
2. $$\begin{aligned}&|f(t,u,u_{\tau },v,v_{\tau })-f(t,u,\tilde{u}_{\tau },v,v_{\tau })|=|P_{1}(t)|\cdot |f_{1}(u_{\tau })-f_{1}(\tilde{u}_{\tau })|\le L_{1}\gamma _{1}(t)|u_{\tau }-\tilde{u}_{\tau }|,\\&|f(t,u,u_{\tau },v,v_{\tau })-f(t,u,u_{\tau },\tilde{v},v_{\tau })|=|P_{2}(t)|\cdot |f_{2}(v)-f_{2}(\tilde{v})|\le L_{2}\gamma _{2}(t)|v-\tilde{v}|,\\&|f(t,u,u_{\tau },v,v_{\tau })-f(t,u,u_{\tau },v,\tilde{v}_{\tau })|=|P_{3}(t)|\cdot |f_{3}(v_{\tau })-f_{3}(\tilde{v}_{\tau })|\le L_{3}\gamma _{3}(t)|v_{\tau }-\tilde{v}_{\tau }|. \end{aligned}$$

Let $\tilde{u}=u+h$, $\tilde{v}=v+k$, using Taylor’s formula,

46 $$g(\tilde{u},\tilde{v})=g(u,v)+\frac{\partial \tilde{g}}{\partial u}h+\frac{\partial \tilde{g}}{\partial v}k,$$

where $$\tilde{g}(u,v)=g(u+\theta h,v+\theta k)$$, $$0<\theta <1$$.

If $g(u,v)=g(\tilde{u},\tilde{v})=0$, (46) results in

$$\tilde{v}-v=\left( \frac{\partial \tilde{g}}{\partial v}\right) ^{-1}\left( \frac{\partial \tilde{g}}{\partial u}\right) (\tilde{u}-u).$$

If (42) is true, then

47 $$|\tilde{v}-v|\le L|\tilde{u}-u|,$$

together with (43), (44), (45), (47), we know that all the conditions of Theorem (16) are satisfied. For example,

$$P_{1}(t)=t, P_{2}(t)=2t^{2}, P_{3}(t)=1+t, \sigma (t)=-(1+2t)^{2},$$

and checking the note from Theorem 1, we just take

$$\begin{aligned} \sigma _{1}(t)=(1+2t)^{2}, f_{1}(u_{\tau })=\sin u_{\tau }, f_{2}(v)=\log (1+v^{2}), f_{3}(v_{\tau })=\cos v_{\tau }. \end{aligned}$$

It can be easily verified by Lagrange mean value theorem that the Lipschitz constant,

$$\begin{aligned} L_{1}=1, L_{2}=\frac{1}{2}, L_{3}=1, L=\sup _{u,v\in \mathbb {R}}\left| \left( \frac{\partial g}{\partial v}\right) ^{-1}\left( \frac{\partial g}{\partial u}\right) \right| . \end{aligned}$$

Let

$$g(u,v)=v-\arctan (1+u),$$

then

$$\frac{\partial g}{\partial u}=-\frac{1}{1+u^{2}},\quad |\frac{\partial g}{\partial u}|\le 1,$$

we get $L=1$ and

$$\begin{aligned}&\frac{1}{2}\sigma _{1}(t)+L_{1}\gamma _{1}(t)+L(L_{2}\gamma _{2}(t)+L_{3}\gamma _{3}(t))-\sigma (t), t\ge 0, \left( \forall t>\frac{1}{\sqrt{2}} actually\right) . \end{aligned}$$

Conditions (1)–(4) are satisfied in the above example. Example 2 and Example 3 show the stable results, while Example 4 shows unstable results.

### Example 2

Let $$x(t), y(t)\in \mathfrak {R}$$, $$f: \mathfrak {R}\times \mathfrak {R}\times \mathfrak {R}\times \mathfrak {R}\times \mathfrak {R}\rightarrow \mathfrak {R}$$,   $$\varphi : \mathfrak {R}\times \mathfrak {R}\times \mathfrak {R}\rightarrow \mathfrak {R}$$.

$$\begin{aligned} x'(t)&= -2x(t)+\frac{1}{e^{2}(1+e^{2})}x(t)y(t-\tau )-\frac{1}{e^{2}(1+e^{2})} x(t-\tau )y(t)\\ 0&=\frac{1}{2}x(t)+\frac{1}{2}x(t-\tau )-y(t)\\ x(t)&= e^{-2t}, \quad -2\tau \le t \le 0\\ y(t)&= \frac{1}{2}(e^{-2t}+e^{-2t+2}), \quad -2\tau \le t\le 0, \end{aligned}$$

Take $$\tau =1$$, then $$\sigma (t)=(-2+\frac{1}{2}e^{-2t})<0, \quad L=\frac{1}{2},\quad K=\frac{1}{2}$$,   $$\sigma _{1}(t)=2$$

$$\begin{aligned} \gamma _{1}(t)=\frac{e^{-2t}}{2e^{2}},\quad \gamma _{2}(t)=\frac{e^{-2t}}{1+e^{2}},\quad \gamma _{3}(t)=\frac{e^{-2t}}{e^{2}(1+e^{2})} \end{aligned}$$

Therefore,

$$\begin{aligned} \frac{1}{2}\sigma _{1}+\gamma _{1}+(L+K)\gamma _{2}+(L+K)\gamma _{3} =1+\frac{3e^{-2t}}{2e^{2}}<-\left( -2+\frac{1}{2}e^{-2t}\right) =\sigma (t). \end{aligned}$$

**Fig. 1:**
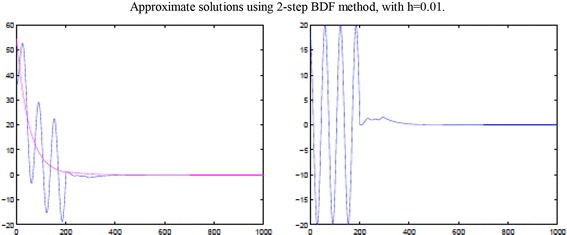
Approximate solutions using 2-step BDF methods with h=0.01

The above results show that all the stability conditions are satisfied, so 2-step BDF methods for the system are stable and asymptotically stable. This can be seen in the following graph (Fig. 1). Table 1 lists errors between numerical solutions and the exact solutions with different step sizes. Table 2 shows numerical results made by implicit Euler method and 2-step BDF method at some time. It can be seen by comparison that BDF method converges much faster than implicit Euler method.

**Table 1:** Errors compared with the exact solution for Example 2

m	h (mh = 1)	$\max |x_n -x(t_n )|$	$\max |y_n -y(t_n )|$
10	0.1	4.2 × 10^−3^	1.75 × 10^−2^
100	0.01	4.83 × 10^−5^	2.03 × 10^−4^
1000	0. 001	4.90 × 10^−7^	2.05 × 10^−6^
2000	0.0005	1.23 × 10^−7^	5.14 × 10^−7^
4000	0.00025	3.06 × 10^−8^	1.29 × 10^−7^
8000	0.000125	7.67 × 10^−9^	3.21 × 10^−8^
16000	0.0000625	1.92 × 10^−9^	8.04 × 10^−9^
10^6^	10^−6^	9.21 × 10^−12^	3.86 × 10^−11^

**Table 2:** *x*_*n*_ and *y*_*n*_ are numerical solutions by implicit Euler method, $\tilde{x}_{n}$ and $\tilde{y}_{n}$ are numerical solutions by BDF method, *x*(*t*_*n*_ and *y*(*t*_*n*_) are exact solutions

*n*	$t_{n} = nh$	*x*_*n*_	*x*_*n*_	$\tilde{x}_n$	$\tilde{y}_n$	*x*(*t*_*n*_)	*y*(*t*_*n*_)
0	0	1.0000	0.0500	1.0000	0.5000	1.0000	0.5000
1	0.1	0.8725	0.4363	0.8607	0.4304	0.8607	0.4304
2	0.2	0.7613	0.3806	0.7403	0.3701	0.7408	0.3704
3	0.3	0.6642	0.3321	0.6371	0.3186	0.6376	0.3188
4	0.4	0.5795	0.2898	0.5484	0.2742	0.5488	0.2744
5	0.5	0.5056	0.2528	0.4720	0.2360	0.4724	0.2362
6	0.6	0.4412	0.2206	0.4063	0.2031	0.4066	0.2033
7	0.7	0.3849	0.1925	0.3497	0.1748	0.3499	0.1750
8	0.8	0.3358	0.1679	0.3010	0.1505	0.3012	0.1506
9	0.9	0.2930	0.1465	0.2590	0.1295	0.2592	0.1296
10	1.0	0.2557	0.1278	0.2230	0.1115	0.2231	0.1116
11	1.1	0.2231	0.1115	0.119	0.0960	0.1920	0.0960
12	1.2	0.1946	0.0973	0.1652	0.0826	0.1653	0.0826
13	1.3	0.1698	0.0849	0.1422	0.0711	0.1423	0.0711
14	1.4	0.1482	0.0741	0.1224	0.0612	0.1225	0.0612
15	1.5	0.1293	0.0646	0.1053	0.0527	0.1054	0.0527
16	1.6	0.1128	0.0564	0.0906	0.0453	0.0907	0.0454
17	1.7	0.0984	0.0492	0.0780	0.0390	0.0781	0.0390
18	1.8	0.0859	0.0429	0.0672	0.0336	0.0672	0.0336
19	1.9	0.0749	0.0375	0.0578	0.0289	0.0578	0.0289
20	2.0	0.0654	0.0327	0.0497	0.0249	0.0498	0.0249

All solutions are on [0, 2] with h = 0.1 and nh = 2

### Example 3

Let $$x(t),\quad y(t)\in \mathfrak {R}\quad f: \mathfrak {R}\times \mathfrak {R}\times \mathfrak {R}\times \mathfrak {R}\times \mathfrak {R}\rightarrow \mathfrak {R},\quad g: \mathfrak {R}\times \mathfrak {R}\rightarrow \mathfrak {R}$$

$$\begin{aligned} x'(t)&=-3x(t)+e^{-6}x(t)y(t-\tau )-e^{-6}x(t-\tau )y(t)\\ 0&=x(t)-2y(t) \end{aligned}$$

Here we take $\tau =2$, its initial functions are

$$x(t)=e^{-3t}, \quad y(t)=\frac{1}{2}e^{-3t}$$

Obviously,

$$\begin{aligned} \sup _{x,y\in \mathfrak {R}}\left| \left( \frac{\partial g}{\partial y}\right) ^{-1}\left( \frac{\partial g}{\partial x}\right) \right| =\frac{1}{2}<\infty \end{aligned}$$

Then it can be found by a simple computation and without losing generality, by taking supremum values

$$\begin{aligned} \sigma&= \sup _{t\ge 0}\left( -3+\frac{1}{2}e^{-3t}\right) =-\frac{5}{2}, \quad \sigma _{1}=\sup _{t\ge 0}\left( 3-\frac{1}{2}e^{-3t}\right) =3,\\ \gamma _{1}&= \sup _{t\ge 0}\left( \frac{1}{2}e^{-3t-3}\right) =\frac{1}{2}e^{-6},\quad \gamma _{2}=\sup _{t\ge 0}(e^{-3t})=1,\qquad \gamma _{3}=\sup _{t\ge 0}(e^{-3t-6})=e^{-6}, \end{aligned}$$

they satisfy

$$\frac{1}{2}\sigma _{1}+\sigma +\gamma _{1}+L(\gamma _{2}+\gamma _{3})<0.$$

**Fig. 2:**
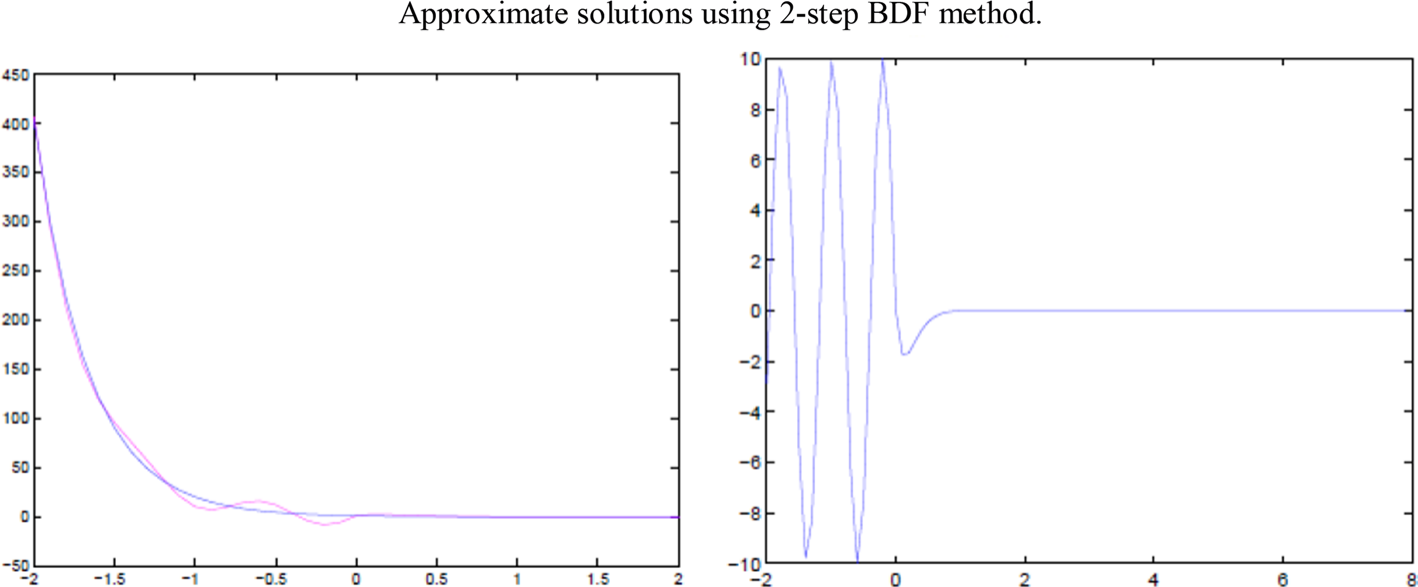
Approximate solutions using 2-step BDF methods

The above results show that all the stability conditions are satisfied, so 2-step BDF methods for the system are stable and asymptotically stable. The simple illustrations are shown in the following graph (Fig. 2) and we can check it by errors of the solutions listed in the following Table 3.

**Table 3:** Errors compared with the exact solution for Example 3

m	h (mh = 2)	$\max |x_n -x(t_n )|$	$\max |y_n -y(t_n )|$
10	0.2	2.49 × 10^−2^	1.25 × 10^−2^
100	0.02	4.22 × 10^−4^	2.11 × 10^−4^
1000	0.002	4.39 × 10^−6^	2.20 × 10^−6^
2000	0.001	1.10 × 10^−6^	5.50 × 10^−7^
4000	0.0005	2.76 × 10^−7^	1.38 × 10^−7^
8000	0.00025	6.89 × 10^−8^	3.45 × 10^−8^
16000	0.000125	1.72 × 10^−9^	8.60 × 10^−10^
10^6^	2 × 10^−6^	5.42 × 10^−12^	2.71 × 10^−12^

### Example 4

$$\begin{aligned} x_{1}'(t)&= (1+x_{2}(t)-\sin t)y(t)+\cos t+\sin t-(x_{2}(t-1)-\sin (t-1))^{2},\\ x_{2}'(t)&= \cos t+x_{2}((t-1)-\sin (t-1),\\ x_{3}'(t)&= y(t)+(x_{2}(t-1)-\sin (t-1))^{2},\\ 0&= (x_{1}(t)-\sin (t))(y(t)-\exp (t)). \end{aligned}$$

Here $u(t)=(x_{1}(t), x_{2}(t), x_{3}(t))^{T}, v(t)=y(t)$. By simple calculation, we get,

$$\begin{aligned} \langle f(t,x,x_{\tau },y,y_{\tau })-f(t,\tilde{x},x_{\tau },y,y_{\tau }), x-\tilde{x}\rangle =\exp (t)(x_{1}-\tilde{x_{1}})(x_{1}-\tilde{x_{1}}), \end{aligned}$$

For the initial data

$$x_{1}(0)=0, x_{2}(0)=0, x_{3}(0)=1, x_{2}(t)=\sin (t), (t\le 0).$$

The solution is

$$x_{1}(t)=\sin (t)-\cos (t)+\exp (t), x_{2}(t)=\sin (t), x_{3}(t)=\exp (t), y(t)=\exp (t).$$

Obviously, this solution is not stable. In fact, we could not find any $\sigma (t)$ satisfies

$$\langle f(t,x,x_{\tau },y,y_{\tau })-f(t,\tilde{x},x_{\tau },y,y_{\tau }),x-\tilde{x} \rangle \le \sigma (t)\Vert x(t)-\tilde{x}{t}\Vert ^{2},$$

Thus conditions of Theorem 1 are not valid.

## Conclusions and notes

While investigating nonlinear 2-delayed differential-algebraic equations, we get two sufficient conditions for the stability and asymptotic stability of 2-step BDF methods and think about how to check the conditions with some example. Although it is quite an early stage, the discussion is a useful enlightenment for differential-algebraic equations with multi-delays in the future. Note the Lipschitz conditions play a key role in this research. Apparently the second inequality in condition (1) seems more nature with the form $\Vert f(t,u,u_{\tau },v,v_{\tau })-f(t,\tilde{u},u_{\tau },v,v_{\tau }) \Vert \le \sigma _{1}(t)\Vert u-\tilde{u}\Vert$, but we find results can also be true and the proofs are analogous.
